# Effect of the ifenprodil administered into rostral anterior cingulate cortex on pain-related aversion in rats with bone cancer pain

**DOI:** 10.1186/s12871-016-0283-1

**Published:** 2016-11-21

**Authors:** Hao Feng, Zhaowei Chen, Gongming Wang, Xin Zhao, Zhonghao Liu

**Affiliations:** 1 Department of Anesthesiology, the Second Hospital of Shandong University, Jinan, China; 2 Plastic surgery, Liaocheng People’s Hospital, Liaocheng, China; 3 Department of Anesthesiology, Shandong Provincial Hospital, Jinan, China; 4 Department of Orthopedics, the Second Hospital of Shandong University, Jinan, Shandong 250033 China

**Keywords:** Bone cancer pain, Aversion, Rostral anterior cingulate cortex, Ifenprodil

## Abstract

**Background:**

To assess the effect of rostral anterior cingulate cortex (rACC) administration with ifenprodil (NR2B receptor blocker) on bone cancer pain (BCP)-related aversion sentiment using the conditioned place avoidance experiments in rats.

**Methods:**

A total of 30 male Wistar rats without place preference were randomly assigned to three groups: control group (Group C, *n* = 10), BCP group (Group P, *n* = 10) and ifenprodil group (Group Ifen, *n* = 10). Three microliter MADB-106 cells were inoculated into right tibia bone marrow cavity in group P and Ifen, while the same dose of normal saline in group C as a control. Ifenprodil was administered into the rACC at the 14th day after inoculation in group Ifen and normal saline in group C and P. Mechanical stimulation pain thresholds of the rats’ right hind paws were measured using Von Frey stimulation method at 1 day before injection of the tumor cells and at 3, 7,10, 12 and 14 days after the injection. The pain-related aversion in rats with BCP was determined by the conditioned place avoidance (CPA) test at 14 days after injection of ifenprodil.

**Results:**

The mechanical stimulation pain thresholds substantially decreased in rats in groups P and Ifen from 10 days to 14 days after the incubation with the MADB-106 cells (*P* < 0.05). There were significant differences in pain thresholds in groups P and Ifen compared to group C at 10, 12 and 14 days after inoculation (*P* < 0.05). The percentage of residence time in chamber A was (30 ± 4%) in group P, which was lower than (52 ± 5%) in group C (*P* < 0.05). After ifenprodil treatment, the percentage time in chamber A increased to (42 ± 5%), which was higher than that in group P and still lower than that in group C (*P* < 0.05).

**Conclusion:**

Ifenprodil administered into rACC as a selective NR2B antagonist can effectively alleviate pain-related aversion sentiment in rats with BCP.

## Background

Bone pain associated with cancer metastasis is the most severe pain among cancer patients and is often resistant to current analgesics. However the biological mechanisms for its onset are not fully understood [1]. Advanced cancer patients with BCP suffer from malignant emotional experience such as anxiety, fear, loneliness and even worldweariness. Physical and psychological harm to patients are more severe than the pain itself [2, 3]. Therefore, alleviation of malignant emotional experience represents an important component of clinical care for improving the quality of life among advanced cancer patients. However, few studies have investigated pain-related aversion sentiment.

Johansen has shown that aversion response could be weakened by formalin injection in plantar after AP-5 (non-selective NMDA receptor antagonist) administration into ACC [4]. In this study, we aimed to investigate the therapeutic effect of ifenprodil as a selective NR2B receptor antagonist administrated into rACC on BCP-related aversion sentiment.

## Methods

### Animals and grouping

The experiments were performed using 30 male Wistar rats, weighing 230–250 g and 5–7 weeks old (from Experimental Animal Research Center of Shandong University). The rats accessed to food and water freely in a controlled temperature (22–24 °C) and humidity (40–60%). My manuscript reporting adhered to the ARRIVE guidelines for the reporting of animal experiments.

Rats were randomly assigned to three groups. Group C (*n* = 10) served as control group. Group P (*n* = 10) received injection of 3 μl MADB-106 cells (4.8 × 10^9^/ml) and served as BCP group. Group Ifen (*n* = 10) received injection of cells and followed by ifenprodil administration into rACC.

### Establishment of rat BCP model

The MADB-106 cells were maintained in Dulbecco’s Modified Eagle’s Medium, supplemented with 10% Fetal bovine serum, 100 unit/ml penicillin, and 100 μg/ml streptomycin; and cultured at 37°C in a humidified atmosphere of 5% CO_2_; and then passaged weekly according to ATCC guidelines. For administration, cells were detached by scraping and then centrifuged at 900 rpm for 3min. The pellet was suspended in Hank’s balanced salt solution and then used for intra-tibial injection. Rat BCP model was prepared according to protocols of previous studies [5, 6]. Three microliter MADB-106 cells were injected into the bone marrow of right tibia except control group under pentobarbital (50 mg/kg) anesthesia. The implantation technique of the intrathecal catheter was modified and performed [7, 8]. The skin was closed with 3–0 silk sutures. After surgery, rats were housed in individual cages. To avoid occlusion of the catheter, 10 μl of normal saline was injected via a catheter on alternate days until the end of the experiment.

### Ifenprodil administration into rACC

rCAA was located using stereotaxic instrument. A polyethylene-10 catheter was inserted under pentobarbital anesthesia. The catheter was passed 2.5 cm caudally through an incision and the external part of the catheter was tunneled subcutaneously. 0.2 g/L ifenprodil of normal saline was injected via a catheter in group Ifen (0.6 μl each side), and the same dose of normal saline was used in groups C and P.

### Pain thresholds analysis

Von Frey instrument was used for analysis of pain thresholds induced by mechanical stimulation at different time points. The rats were acclimated to the environment for 20min, and the plantar of their paws were stimulated. Value of the reaction to stimulation within 20s was regarded as pain thresholds. The measurement was repeated for three times with 5min interval, and the average value was taken. The procedure was the same as in contralateral side. The rats were kept in the same status during the process.

### CPA analysis

CPA analysis began at 12 days after injection of cells when pain to stimulation was obvious. Training box consisted of opaque rooms A (condition training room) and B (control room) placed side by side. There were significant differences in visual, tactile and olfactory information. Room A was characterized by black and white vertical stripes on the wall, rough floor and interior drops of 1% acetic acid as the odorant, and room B by black and white horizontal stripes on the wall, smooth floor and 1% cinnamon water. Recording system could automatically record residence time of rats in each room. On first day (pre-training), the rats with BCP explored the box for 20min and could freely enter the room A or B, and time in each room was record. On second day (training period), operated hind paws were stimulated with Von Frey filaments (60 g) every 30s to induce emotional responses when the rats entered room A, while stimulation was given to lateral hind paws when the rats entered room B. On the last day (test period), the time in each room was recorded within 20min, no stimulation was given. Time percentage in room A was calculated.

### Ifenprodil injection site in rACC

Microinjection sites of ifenprodil marked with 0.6 μl saturated Chicago sky blue 6 B were injected into rACC after CPA experiment. Anterior cingulated cortex tissue was obtained and dehydrated using graded sucrose at 4 °C after perfusion with 4% formaldehyde (pH = 7.2). Microinjection sites and diffusion range were analyzed with Paxinos and Watson stereotaxic atlas after consecutive coronal slices (35 μm) [8]. Sites deviated from rACC were not included in the results.

### Statistical analysis

Statistical analysis was done using SPSS 17.0 statistical software. Measurement data was expressed as mean ± standard deviation, different time points in group were compared using repeated measures analysis of variance, one-way analysis of variance was used between groups. *P* < 0.05 was considered statistically significant.

## Results

### Pain thresholds changes of operated jaw in rats

Pain thresholds of rats’ operated jaw decreased significantly in groups P and Ifen compared to group C at 10 days after injection of MADB 106 cells and remained low at 12 and 14 days (*P* < 0.05). There was no changes in group C. Between groups, pain thresholds was lower in groups P and Ifen at 10, 12 and 14 days than that in group C (*P* < 0.05) (Table 1).

**Table 1:** Pain thresholds of operated jaw in rats

Group	Preoperation	Postoperation (d)				
		3	7	10	12	14
Group C (*n* = 9)	12.0 ± 2.4	11.6 ± 2.8	12.2 ± 2.6	12.4 ± 2.2	12.0 ± 2.1	11.8 ± 2.1
Group P (*n* = 8)	12.2 ± 2.1	10.5 ± 2.2	10.2 ± 2.6	7.3 ± 2.1^ab^	5.8 ± 1.8^ab^	5.2 ± 1.4^ab^
Group Ifen (*n* = 9)	11.9 ± 1.8	11.2 ± 2.2	10.8 ± 2.4	7.8 ± 2.3^ab^	5.4 ± 1.2^ab^	5.0 ± 1.8^ab^

^a^ *P < 0.05*,*vs* group C, ^b^ *P < 0.05 vs* postoperation

### Room residence time in different groups

Residence time in room A was (30 ± 4%) in group P, shorter than (52 ± 5%) in group C (*P* < 0.05). In group Ifen, residence time in room A was decreased to (42 ± 5%), which longer than that in group P and still shorter than that in group C (*P* < 0.05) (Table 2).

**Table 2:** Time percentage in room A in all rats

Group	Time percentage (%)
Group C (*n* = 9)	52 ± 5
Group P (*n* = 8)	30 ± 4^a^
Group Ifen (*n* = 9)	42 ± 5^ab^

^a^ *P < 0.05 vs* group C, ^b^ *P < 0.05 vs* group P

### Histological examination of Ifenprodil injection site

**Fig. 1:**
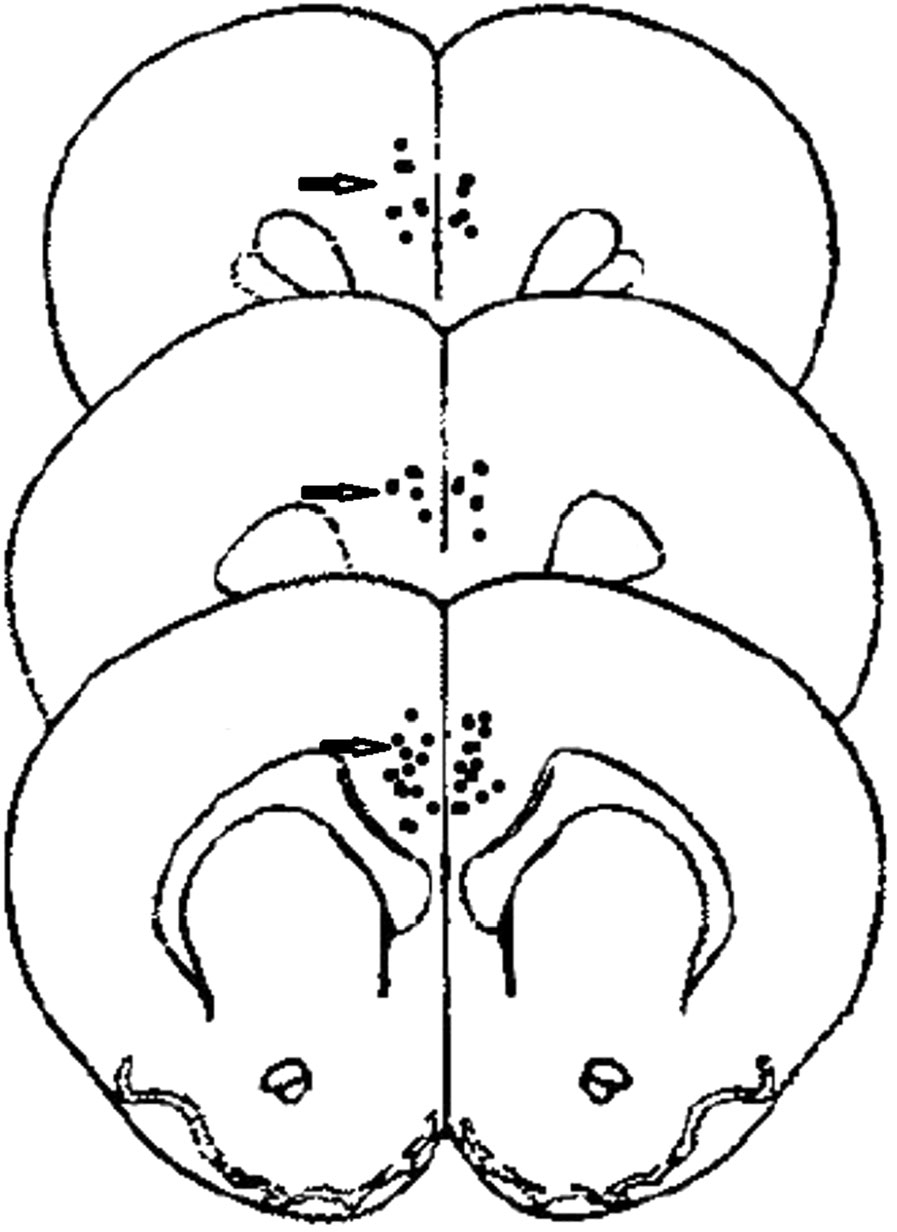
Histological examination of injection sites of ifenprodil in rACC (*arrows* show the sites and diffusion range)

The results showed that one in group C, one in group Ifen and two in group P were excluded because of incorrect administration. Ifenprodil injection sites and the diffusion range are shown in Fig. 1.

## Discussion

Bone metastasis-induced pain manifests as spontaneous pain, hyperalgesia, and allodynia [9]. Pain that is severe enough to compromise daily life affects about 36–50% of cancer patients [10]. Therefore, studies about BCP are important in clinical practice. MADB-106 breast cancer cell line of rat was from breast cancer lung metastasis (MADB-100) and cultured with Fish 344 rat. Applications of MADB-106 cells in rat model with BCP have achieved high success rate. In our study, the rats had significant hyperalgesia at 10 days after injection of MADB-106.

Rats exhibit avoidance behavior when exposed to painful stimuli, and this pain-related information is stored in the memory. Rats can avoid noxious stimuli through forecast because of the bad memories of painful emotional experience when the pain stimuli appear again. Thus, avoidance behavior of rats to painful stimuli may reflect pain aversion. Our results showed avoidance of rats with BCP from room A the next day after received mechanical stimulation, and this avoidance behavior in rats resulted from bad memories of pain experience. Thus, residence time in room A could reflect pain related aversion in rats with BCP.

ACC neurons are involved in emotional processing of pain, making it an attractive target for therapeutic intervention on pain-related aversion [11, 12]. Ifenprodil injection was used in this study to investigate the therapeutic effect on pain-related aversion, and we used saturated Chicago sky blue 6B to confirm injection sites in rACC. Residence time in room A became longer after the rats with BCP received ifenprodil injection into rACC, suggesting that pain related aversion alleviated. Clinical observations suggested that anxiety, depression and other negative emotions of patients with intractable pain could be significantly alleviated after ACC and the surrounding cortex were removed. Rainville found that pain-related aversion became worse when the stimulus intensity increased, or ACC neurons excitement level increased [13]. Microinjection of non-selective NMDA receptor antagonist AP-5 into ACC could block formalin-induced conditioned place avoidance [4]. This indicated that NMDA receptors in ACC neurone play an important role in the generation and maintenance of chronic pain to vicious emotional experience. NMDA receptor consists of NR1 subunit and NR2A/NR2B subunit in amygdala and prefrontal cortex. Chronic inflammatory pain response increased with expression of NR2B in forebrain [14], and expression of NR2B in ACC upregulated after plantar subcutaneous injection of CFA to produce inflammatory pain [15]. Taken together with our study results, these data suggest that NR2B receptor in rACC neurons plays an important role in pain-related aversion and can be a potential therapeutic target for treatment of BCP associated with disgust.

## Conclusions

Injection of selective NR2B receptor antagonist ifenprodil into rACC regions could significantly reduce aversion associated with BCP in rats. Our study provides a new approach for clinical treatment and research of BCP-related aversion.

## Abbreviations

ATCC: American type culture collection
BCP: Bone cancer pain
CPA: Conditioned place avoidance
NMDA: N-methyl-D-aspartic acid receptor
rACC: rostral anterior cingulate cortex
